# Hospitalizations due to respiratory failure in patients with Amyotrophic Lateral Sclerosis and their impact on survival: a population-based cohort study

**DOI:** 10.1186/s12890-016-0297-y

**Published:** 2016-11-03

**Authors:** Federica Edith Pisa, Giancarlo Logroscino, Paolo Giacomelli Battiston, Fabio Barbone

**Affiliations:** 1 Institute of Hygiene and Clinical Epidemiology, University Hospital of Udine, Via Colugna 50, Udine, 33100 Italy; 2 Neurodegenerative Diseases Unit, Department of Basic Medicine Neuroscience and Sense Organs, Department of Clinical Research in Neurology of the University of Bari at “Pia Fondazione Card G.Panico” Hospital Tricase, Lecce, University of Bari, Bari, Italy; 3 Department of Biological and Medical Sciences, University of Udine, Udine, Italy; 4 Department of Medical Sciences, University of Trieste, Trieste, Italy

**Keywords:** Amyotrophic Lateral Sclerosis, Hospital utilization, Hospitalization, Respiratory failure, Non-invasive ventilation, Tracheostomy, Mechanical ventilation, Survival, Cohort studies, Database research

## Abstract

**Background:**

Respiratory failure, infections and aspiration pneumonia, are the main causes of morbidity and mortality in Amyotrophic Lateral Sclerosis (ALS). In a population-based cohort, we assessed (a) hospital utilization and (b) impact of hospitalization for respiratory failure on survival.

**Methods:**

All patients with incident ALS in Friuli Venezia Giulia region, Italy, from 2002 to 2009, were identified through multiple sources. Diagnosis was validated through clinical documentation review. For each patient, we extracted the records of all hospitalizations after ALS diagnosis from the regional hospitalization database. Cox proportional hazards model survival Hazard Ratio (HR), with 95 % Confidence Interval (95 % CI), was calculated.

**Results:**

Out of 262 patients, 98.1 % had at least 1 and 58.0 % ≥3 hospitalizations. Emergency admissions occurred in 77.5 % of patients and a diagnosis of respiratory failure in 55.0 %. Patients underwent a total of 885 hospitalizations. The leading diagnosis was respiratory failure (31.6 % of hospitalizations). This diagnosis occurred most frequently in emergency (45.6 %) than in elective admissions (26.4 %). The second leading diagnosis was pneumonia (14.2 %), 24.9 and 6.3 % respectively. The leading procedure was mechanical ventilation (18.4 %), performed in 29.9 % of emergency and in 12.4 % of elective admissions. After adjustment for site of onset, age and diagnostic delay, a first hospitalization for respiratory failure had a strong adverse effect on survival (HR 4.00; 95 % CI 3.00; 5.34).

**Conclusions:**

Respiratory failure, pneumonia and aspiration pneumonia were major determinants of hospitalizations and emergency admissions and often dealt with in emergency admissions. A first hospitalization for respiratory failure had a strong adverse effect on survival. Strategies to improve home management of respiratory conditions in patients with ALS and to optimize hospital care utilization are needed.

**Electronic supplementary material:**

The online version of this article (doi:10.1186/s12890-016-0297-y) contains supplementary material, which is available to authorized users.

## Background

During the course of disease, patients with Amyotrophic Lateral Sclerosis (ALS) experience a progressive loss of muscular function leading invariably to disability, paralysis and death. In European population-based studies, median survival time was between 25 and 30 months from disease onset [1]. Respiratory failure and complications such as infections and aspiration pneumonia are the main causes of morbidity and mortality. Respiratory function is strongly related to ALS outcome [2]. In the early phase of disease, Sniff Nasal Inspiratory Pressure is a strong predictor of death or tracheostomy within 1 year of follow-up [3]. Decreased Forced Vital Capacity due to muscle weakness has been associated with an increased risk of short-term mortality [4]. In patients with early respiratory failure, non-invasive ventilation (NIV) is recommended [5, 6]. NIV prolongs survival and may improve patients’ quality of life [7]. Recommendations include advance directives and planning of respiratory failure management to avoid emergency invasive ventilation [5]. Invasive mechanical ventilation can prolong survival but has uncertain impact on the quality of life of patients and caregivers [5, 8]. The prevalence of invasive ventilation varies greatly between countries [9] and within Italy from 10.6 % [10] to 24.5 % [11], as reported by population-based studies.

Hospital care utilization is common in patients with ALS and represents the third main contributor to costs of care, after home care and ventilation [12]. The proportion of patients hospitalized decreased from 80 % in 2000 to less than 40 % in 2012 [13]. Evaluating hospital admissions with a diagnostic code for ALS in large US administrative databases, two studies reported that emergency admissions were common while only about 20 % were elective [14, 15]. The main reasons for hospitalization were respiratory failure and pneumonia. Patients admitted for respiratory failure showed a 5-fold increased risk of dying in hospital (odds ratio of death: 5.03; 95 % CI 4.57–5.54); in-hospital mortality was 25.4 % in the subgroup undergoing CPAP/BiPAP and 30 % in the subgroup undergoing mechanical ventilation ˂96 h [14].

In other respiratory diseases such as idiopathic pulmonary fibrosis, early and respiratory-related hospital admissions have been associated with increased all-cause mortality [16]. In patients with ALS, the effect of hospitalizations for respiratory conditions on overall survival has not been evaluated.

Several studies suggest that the modality of health care provision for patients with ALS has a relevant impact on quality of life and use of healthcare facilities. Patients followed up by multidisciplinary centers had lower in-hospital mortality and shorter length of stay [10]. Community care management and coordination led to a decrease in overall and emergency non-planned hospitalizations [13]. Telemonitoring of home NIV reduced hospital admissions and emergency room visits [17].

This population-based cohort study of incident ALS cases in Friuli Venezia Giulia (FVG) region, Northeastern Italy, from 2002 to 2009, aims to assess: (a) hospitalizations after the diagnosis of ALS, focusing on admissions and procedures related to respiratory problems and (b) the impact of hospitalizations for respiratory failure on survival.

## Methods

### Study population

The study was conducted in FVG resident population, approximately 1,200,000 inhabitants [18].

### Data sources

All FVG residents are registered with the Regional Health System, providing universal access to care. Their use of health services is registered in computerized databases. Anonymized individual records can be linked by a unique personal identifier. For this study the database of hospitalizations was used. This database records data of all hospitalizations of residents in hospitals of FVG and of other Italian regions. Day-hospital admissions, where the patient was admitted and discharged within 24 h, are also included. For each hospitalization, the following information is registered: dates of admission and discharge, type of admission (emergency, elective or day hospital), one primary and up to five secondary discharge diagnostic codes and up to six codes for procedures, if hospitalization ended with death and date of death. Emergency admissions are those not planned in advance but occurring abruptly due to clinical need.

Diagnoses and procedures are coded according to the International Classification of Diseases, ninth revision, clinical modification (ICD-9-CM) coding system.

### Study cohort

The cohort included any resident with an incident diagnosis of ALS from January 1st, 2002 to December 31, 2009. The ascertainment of cases has been described elsewhere [19]. Multiple sources, including database of hospitalizations, electronic medical records and archives of Neurology departments, were used to detect patients diagnosed during in- or out-patient medical encounters. ALS diagnosis was validated through clinical documentation review. For each detected case, four neurologists independently reviewed clinical documentation for any hospitalization or relevant healthcare encounter up to December 31, 2009, to confirm the diagnosis. ALS confirmation was based on information relative to the entire follow-up and required the following criteria to be met: (a) ALS diagnosis explicitly written by a neurologist and/or (b) prescription for Riluzole in the therapeutic plan. All patients with confirmed ALS and a diagnosis of possible, probable or definite ALS according to El Escorial criteria [20, 21] were included.

### Data collection

Information on date of diagnosis and site of onset was abstracted from clinical documentation. The date of diagnosis was defined as (a) the date of discharge when ALS was diagnosed or confirmed during a hospitalization (the diagnosis was written by the physician on the discharge letter) or (b) the date of the examination, when ALS was diagnosed during an outpatient examination. We extracted the records of all hospitalizations from January 1st, 2000 to December 31, 2011, information on residential history (to account for potential emigration from the region) and date of death, up to December 31, 2011.

Hospitalizations after the diagnosis of ALS where those with date of admission after the date of diagnosis. Vital status of patients at the end of follow-up (December 31, 2011) was ascertained through record linkage with the mortality database.

### Statistical analysis

Taking the patient as statistical unit, we performed a descriptive analysis of their characteristics and hospitalizations according to ALS site of onset. Frequencies were compared through the chi square test. Kaplan Mayer product-limit survival function was used to calculate survival times, overall and after the first hospitalization (a) for respiratory failure, both as primary or any secondary code, (b) tracheostomy or (c) mechanical ventilation (MV).

Survival Hazard Ratios (HR), with 95 % Confidence Interval (95 % CI), were calculated through Cox proportional hazards model. The following terms were assessed in univariate analysis: having a first hospitalization for respiratory failure after the diagnosis of ALS, type of onset, sex, age at disease onset, diagnostic delay (lag time between the onset of symptoms and diagnosis), Charlson Comorbidity Index and having a hospitalization for respiratory failure before the diagnosis of ALS. The first hospitalization for respiratory failure after the diagnosis of ALS was defined as the first hospitalization after the date of diagnosis with one of the following primary or any secondary ICD-9-CM codes: 518.81 acute respiratory failure, 518.83 chronic respiratory failure, 518.84 acute on chronic respiratory failure and 518.82 unidentified respiratory failure. This variable was time‐dependent and was therefore included in the model using the counting process method [22].

A hospitalization for respiratory failure before the diagnosis of ALS was defined as occurred any time before the date of diagnosis with one of the above reported primary or any secondary ICD-9-CM codes.

Taking the hospitalization as the statistical unit, we performed a descriptive analysis of diagnoses and procedures according to the type of admission (emergency, elective or day-hospital).

Statistical analysis was performed using SAS© statistical package 9.2 (SAS Institute Inc., Cary, N.C., USA).

## Results

A total of 262 patients, 50.4 % men, 49.6 % aged ≥ 68 years at diagnosis, were identified (Table 1). The site of onset was spinal in 50.7 % and bulbar in 34.0 %. Information on the site of onset was missing in 15.3 % (*N* = 40).

**Table 1:**
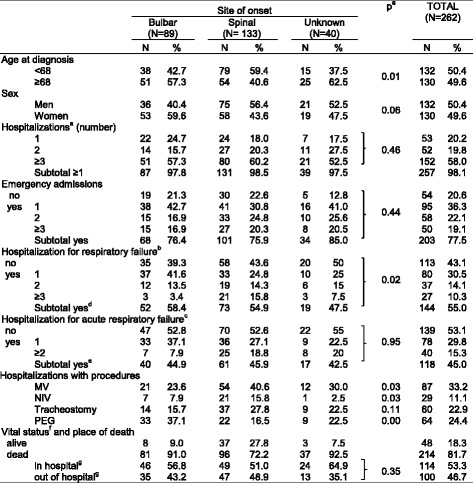
Number and distribution of patients with ALS by selected characteristics and hospitalizations after the diagnosis of ALS

^a^Chi square test p comparing proportions between patients with bulbar, spinal and unknown site of onset

^b^Excludes one patient who did not have any hospitalization after the diagnosis of ALS and four patients who died during the hospitalization in which the diagnosis of ALS was made

^c^At least one hospitalization after the diagnosis of ALS with discharge code for respiratory failure (ICD-9-CM codes 518.81, 518.82, 518.83, 518.84) in any position

^d^Subtotal of patients with at least one hospitalization with discharge code for respiratory failure

^e^Subtotal of patients with at least one hospitalization with discharge code for acute respiratory failure

^f^Dead or alive at the end of follow up, December 31, 2011

^g^The denominator of percentages is the total number of deceased patients

Four patients died during the hospitalization where the diagnosis was made and were excluded from the analysis. Only one patient did not have any hospitalization after the diagnosis, 98.1 % (*N* = 257) were hospitalized at least once after the diagnosis and 58.0 % three or more times. More than 75.0 % had at least one emergency admission and 41.2 % two or more.

More than 50 % were hospitalized at least once for respiratory failure and 45.0 % for acute respiratory failure. Hospitalizations for respiratory failure were more common in patients with bulbar onset (58.4 %), than in those with spinal (54.9 %) or unknown (47.5 %) onset (*p* = 0.02). One third of patients underwent MV, more frequently those with spinal onset (40.6 %) than with bulbar (23.6 %) or unknown onset (30.0 %) (*p* = 0.03). Overall, 22.9 % underwent tracheostomy, more frequently those with spinal onset (27.8 %). Only 11.1 % (*N* = 29) underwent in-hospital NIV.

By the end of follow-up, 81.7 % of patients were deceased; 53.3 % of them died in hospital. During follow-up, the cohort had 885 hospitalizations (Table 2). Emergency admissions accounted for 47.6 %. Median length of stay was 8 days (Inter Quartile Range IRQ 4; 15) in elective and 8 days (IQR 4; 17) in emergency admissions (data not shown).

**Table 2:** Number and distribution of the most common discharge diagnoses, by type of admission

	Type of admission															
	Emergency (*N* = 421)				Elective (*N* = 299)				Day hospital (*N* = 165)				Overall (*N*=885)			
Discharge diagnosis (ICD-9-CM codes)	Primary code^a^		Any code^b^		Primary code^a^		Any code^b^		Primary code^a^		Any code^b^		Primary code^a^		Any code^b^	
	N	(%)	N	%	N	%	N	%	N	%	N	%	N	%	N	%
ALS (335.20)	112	(26.6)	335	(79.6)	154	(51.5)	233	(77.9)	122	(73.9)	132	(80.0)	388	(43.8)	700	(79.1)
Respiratory failure (518.81-.84)	117	(27.8)	192	(45.6)	49	(16.4)	79	(26.4)	6	(3.6)	9	(5.4)	172	(19.4)	280	(31.6)
Acute (518.81)	93	(22.1)	138	(32.8)	44	(14.7)	59	(19.7)	-		2	(1.2)	137	(15.5)	199	(22.5)
Chronic (518.83)	3	(0.7)	9	(2.1)	2	(0.7)	15	(5.0)	6	(3.6)	6	(3.6)	11	(1.2)	30	(3.4)
Acute on chronic (518.84)	12	(2.8)	25	(5.9)	1	(0.3)	2	(0.7)	-		-		13	(1.5)	27	(3.0)
Unidentified (518.82)	9	(2.1)	21	(5.0)	2	(0.7)	3	(1.0)	-		1	(0.6)	11	(1.2)	25	(2.8)
Diseases of the respiratory system (460-519)	71	(16.9)	156	(37.0)	7	(2.3)	30	(10.0)	-		5	(3.0)	78	(8.8)	191	(21.6)
Pneumonia and influenza (480-487)	44	(10.4)	105	(24.9)	5	(1.7)	19	(6.3)	-		2	(1.2)	49	(5.5)	126	(14.2)
Aspiration pneumonia (507.0)	10	(2.4)	18	(4.3)	-		1	(0.3)	-		-		10	(1.1)	19	(2.1)

^a^Number and percentage (%) of hospitalizations with the corresponding discharge diagnosis as primary code

^b^Number and percentage (%) of hospitalizations with the corresponding discharge diagnosis as primary or any secondary code

Respiratory failure was the most common discharge diagnosis, occurring as primary code in 19.4 % and in any position in 31.6 % hospitalizations, followed by pneumonia (5.5 and 14.2 %, respectively) and aspiration pneumonia (1.0 and 2.1 %, respectively).

Respiratory failure, acute respiratory failure and pneumonia were more frequent in emergency admissions, occurring as primary and/or secondary code in 45.6, 32.8 and 24.9 % hospitalizations, respectively. Conversely, acute respiratory failure or pneumonia were uncommon in day hospital admissions (each 1.2 %). Aspiration pneumonia occurred almost exclusively in emergency admissions.

More than 80 % of the 114 patients who died in hospital, died in emergency hospitalizations. In hospitalizations ending with death, respiratory failure (58.8 %) and diseases of the respiratory system (43.9 %) were the most common codes (Additional file 1: Table S1).

The most common procedure was MV, performed in 18.4 % hospitalizations and more frequently (13.2 %) lasting ≥96 h, followed by tracheostomy (7.1 %) and NIV (3.9 %) (Table 3). MV was more common in emergency (29.9 %) than in elective (12.4 %) admissions, as well as NIV (5.0 % vs. 2.0 %) and tracheostomy (12.6 % vs. 3.3 %). Conversely, gastro–or entero–stomies were performed more frequently in elective (13.0 %) admissions.

**Table 3:** Number and distribution of procedures, by type of admission

Procedures (ICD-9-CM codes) ^a^	Type of admission						Overall (*N* = 885)	
	Emergency (*N* = 421)		Elective (*N* = 299)		Day hospital (*N* = 165)			
	N	%	N	%	N	%	N	%
Respiratory function								
Mechanical ventilation (96.7–96.72)	126	29.9	37	12.4	-	-	163	18.4
≥ 96 h (96.72)	93	22.1	24	8.0	-	-	117	13.2
< 96 h (96.71)	17	4.0	4	1.3	-	-	21	2.3
Unspecified duration	16	3.8	9	3.0	-	-	25	2.8
Tracheostomy (31.1–31.2)	53	12.6	10	3.3	-	-	63	7.1
Non invasive ventilation (93.90)	21	5.0	6	2.0	8	4.8	35	3.9
IPPB (93.91)	6	1.4	7	2.3	-	-	13	1.5
Other respiratory therapy (93.94, 93.96, 93.97, 93.99)	43	10.2	8	2.7	-	-	51	5.8
Measurement of arterial blood gases (89.65)	79	18.8	40	13.3	7	4.2	126	14.2
Nutrition								
Gastro- or entero- stomy	40	9.5	39	13.0	7	4.2	86	9.7
PEG^b^ (43.11)	32	7.6	29	9.6	7	4.2	68	7.6
PEJ^c^ (46.32)	5	1.1	6	2.0	-	-	11	1.2
Other gastrostomy (43.19)	3	0.7	4	1.3	-	-	7	0.7
Diagnostic procedures								
Electromyography (93.08)	16	3.8	47	15.7	49	29.7	112	12.6
MRI^d^ of brain, brain stem and spinal canal (88.91, 88.93)	7	1.6	24	8.0	29	17.6	60	6.8
Spinal tap (03.31)	4	0.9	10	3.3	5	3.0	19	2.1
Bronchoscopy (33.21)	52	12.3	16	5.3	1	0.6	69	7.7
Open biopsy of soft tissue (83.21)	1	0.2	14	4.6	-	-	15	1.6

^a^ ICD-9-CM codes for procedures in any position

^b^ Percutaneous endoscopic gastrostomy

^c^ Percutaneous endoscopic jejunostom

^d^ Magnetic resonance imaging

Diagnostic procedures, such as electromyography (12.6 %) and magnetic resonance imaging (MRI) (6.8 %), were most common in day-hospital admissions (29.7 and 17.6 %, respectively).

**Fig. 1:**
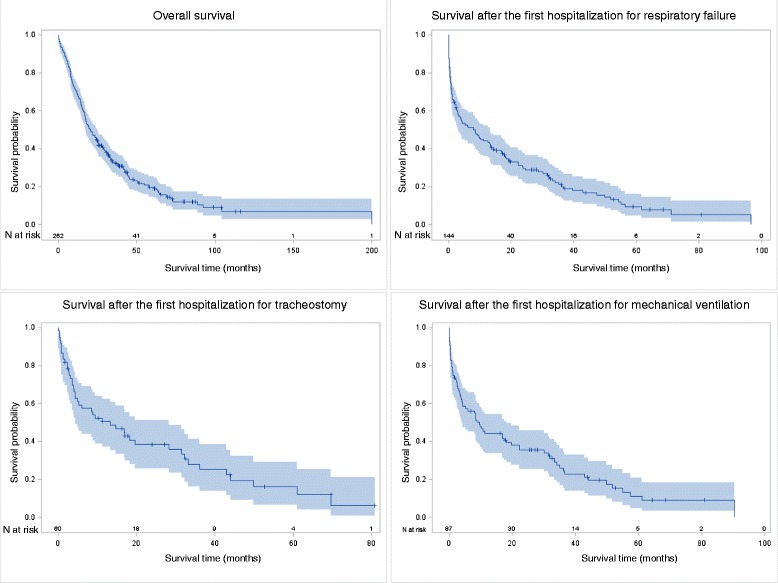
Kaplan-Mayer plot with 95 % Hall-Wellnerbands

The overall median survival of the entire cohort was 20.5 months (95 % CI 17.2; 24.9) (Fig. 1). After the first hospitalization for respiratory failure, median survival time was 8.0 months (95 % CI 3.1; 13.2) and after the first hospitalization with MV 9.7 months (95 % CI 4.1–18.2).

The first hospitalization with diagnosis of respiratory failure occurred on average 32.2 months (Standard Deviation SD 2.0; median 23.8 months) and with MV 21.0 months (SD 1.9; median 15.8 months) after the diagnosis (data not shown).

When simultaneously adjusting for site of onset, age and diagnostic delay, the first hospitalization for respiratory failure was associated with a fourfold increased risk of death (HR 4.00, 95 % CI 3.00; 5.34) (Table 4). Adding a term for Charlson Index or hospitalizations before diagnosis (a total of 16 patients were hospitalized before diagnosis) did not change the results (Additional file 1: Table S2).

**Table 4:** Relation between mortality, hospitalizations for respiratory failure and clinical characteristics. Hazard Ratio (HR), with 95 % Confidence Interval (95 % CI)

	Univariate			Multivariate^a^		
	*p*	HR	95 % CI	*p*	HR	95 % CI
Hospitalization for respiratory failure						
No^b^		1.00	- -		1.00	- -
Yes	<0.0001	3.52	2.65; 4.67	<0.0001	4.00	3.00; 5.34
Site of onset						
Spinal^b^		1.00	- -		1.00	- -
Bulbar	0.0005	1.71	1.27; 2.32	0.0791	1.32	0.97; 1.81
Missing	0.0084	1.69	1.14; 2.49	0.2113	1.31	0.86; 2.01
Age (years)						
< 68^b^		1.00	- -		1.00	- -
≥ 68	<0.0001	2.05	1.56; 2.69	<0.0001	2.50	1.86; 3.36
Diagnostic delay						
≥ median^b, c^		1.00	- -		1.00	- -
< median^c^	0.0065	1.81	1.18; 2.77	0.0004	2.23	1.43; 3.47
Unknown	0.1095	1.34	0.94; 1.91	0.0459	1.48	1.01; 2.19

^a^ site of onset, age, diagnostic delay

^b^ Reference category

^c^ Median: 276 days

## Discussion

In our population-based cohort, almost all ALS patients were hospitalized at least once and more than 75 % twice or more. More than half of patients were hospitalized at least once for respiratory failure, mostly acute. Among all hospitalizations, respiratory failure was the leading discharge diagnosis, followed by other respiratory conditions and pneumonia including aspiration pneumonia. These results are consistent with those reported by two studies evaluating hospital admissions with a diagnostic code for ALS in US administrative databases [14, 15]. Respiratory failure and other respiratory conditions were more frequent in emergency than in elective admissions. Emergency admissions were common in patients with ALS: between 40 and 54 % of patients were admitted through the emergency room [14, 15, 23].

The leading procedures were those related to respiratory function, in particular MV was performed in one third of patients, and tracheostomy in more than 20 %, similarly to [11, 24] or higher than [10] other Italian studies. We do not have information to quantify the percentage of patients who refused tracheostomy or other procedures.

Only 11.1 % of patients underwent NIV during an in-hospital stay. The prevalence of NIV was 47.7 % in another Italian study collecting information from physicians providing patient care [11]. As hospital administrative data capture only in-hospital procedures, information on NIV performed out of hospital is lacking. Our results, therefore, do not represent the overall percentage of patients undergoing NIV but only those receiving it in-hospital. Patients with bulbar onset received NIV, MV and tracheostomy less frequently than those with spinal onset, consistently with other studies [11].

Tracheostomies, MV and in-hospital NIV were performed mostly in emergency non-programmed hospitalizations. Respiratory conditions were treated often in emergency admissions, contrary to recommendations [5, 6]. This result may indicate poor planning of respiratory care and highlights an area for improvement of care of ALS patients. Consistently, in a recent study more than two-thirds of tracheostomies were placed in emergency [25].

Hospitalizations for respiratory failure increased the risk of in-hospital mortality from more than three-fold [15] to five-fold [14]. The longitudinal design of our study, with follow-up performed trough record-linkage with hospitalization and mortality records, allowed us to assess the impact of the first hospitalization for respiratory failure after the diagnosis of ALS on survival time and risk of death, including deaths occurred out of hospital after discharge. After the first hospitalization for respiratory failure, survival time was 8.0 months and after the first hospitalization with MV 9.7 months, shorter than in other population-based Italian studies, respectively of 19 months (95 % CI, 7–43) [11] and 21 months [10]. The first hospitalization for respiratory failure was a strong and independent negative prognostic factor.

The median duration of hospital stay was 8 days, in line with the results of a prior Italian study (mean 10.3 days) [26] but longer than in a French study (mean 3.3 days) [13]. In the USA, the mean length of stay varied from 8.4 days [15] to 5 days [14].

Consistently with our results, in-hospital mortality was not associated with Charlson comorbidity score [14, 15]. In our study, a score ≥ 3 appeared protective. Our results did not vary substantially when the term for Charlson Index score was removed from the model. The Charlson Index assigns the highest weights to conditions such as malignancies, diabetes with organ damage or moderate to severe renal disease, and may not capture clinical conditions that specifically influence the risk of death in patients with ALS, such as malnutrition [27] or respiratory problems. Comorbidities such as coronary heart disease, history of myocardial infarction, diabetes mellitus, seem to have no effect on the survival of ALS patients [28]. Moreover, these patients had less cardiovascular comorbidity and risk factors compared to the general population [28–30].

Nutritional deficiency occurred only in a small percentage of hospitalizations, contrary to other studies [14, 15]. Nutritional procedures and gastro-enterostomies were performed mostly during elective hospitalizations. Diagnostic procedures were most common in day-hospital admissions. These findings suggest that the diagnostic pathway as well as nutritional support were well planned.

In our study, more than half of deceased patients died in hospital. This high percentage calls for improved advance terminal care planning.

In the region FVG, patients with ALS are offered clinical follow-up at the two University Hospitals of the Region. They are referred to outpatient respiratory clinics for assessment and follow-up of respiratory conditions. At the time of the study conduct, however, there was no common regional guideline for respiratory follow-up and home care nor for timing of initiation and methods of ventilation. Therefore, the follow-up of ALS patients was probably not uniform.

Integrated health care provision, including follow-up and home care of respiratory problems, has improved patients’ quality of life and optimized hospital utilization, reducing emergency admissions [13, 17]. Our study therefore highlighted important areas of improvement in supportive care of patients with ALS.

### Strengths

The main strength of our study is its population-based and longitudinal design.

This cohort included all incident cases in a well-defined time period and geographical area. The ascertainment of cases was complete, as suggested by incidence rates within the range of other European registries [19]. All study subjects were confirmed incident cases of ALS. Diagnosis was made according to consensus criteria and validated by an expert neurologist.

The probability of emergency gastrostomies varied according to ethnicity and health insurance of patients [25]. Our study was conducted in a universal health care system. All members of the cohort are residents of FVG and have the same criteria for access to hospital care, irrespectively of socio-economical status. The results are therefore not influenced by differential access to hospital care.

Hospitalization episodes were obtained from health administrative databases, an objective source. All episodes were captured, as the database records all hospitalizations occurred in public and private hospitals of FVG and of other Italian regions.

### Limitations

Factors such as living without a partner [31], area of residency [32], nutritional status at diagnosis or during the course of disease [33] as well as a diagnosis of frontotemporal dementia [31], have an unfavorable prognostic role in ALS. Information on clinical, psychosocial or environmental factors were not recorded in our data and we could not assess their role.

As we lack information on preferences of patients and families, the percentage of patients offered NIV/MV in advance and the percentage of those who accepted could not be calculated.

## Conclusions

In this population-based cohort of patients with ALS, hospitalizations were very common. Respiratory failure, pneumonia and aspiration pneumonia were the leading causes for hospital care utilization. Respiratory conditions were often dealt with in emergency admissions. Hospitalizations for respiratory failure had a strong independent negative effect on survival.

This study identified important areas for improvement in the care of patients with ALS. Strategies such as community-based interventions and care may improve the management of respiratory problems and reduce unnecessary emergency admissions. Improved advance terminal care planning may reduce the percentage of in-hospital deaths.

More research is needed to assess the relation between patients’ characteristics, such as socio-demographic factors, presence of support or area of residency, and hospital care utilization. Such studies could identify key factors in designing and implementing interventions aimed at improving the management of respiratory conditions in patients with ALS.

## Additional file

Additional file 1: Table S1. Number and distribution of discharge diagnoses by type of admission, in hospitalizations ending with death. **Table S2.** Relation between mortality, hospitalizations for respiratory failure and clinical characteristics. Hazard Ratio (HR), with 95 % Confidence Interval (95 % CI). (DOCX 30 kb)

## Abbreviations

ALS: Amyotrophic Lateral Sclerosis
BiPAP: Bilevel Positive Airway Pressure
CPAP: Continuous Positive Airway Pressure
FVG: Friuli Venezia Giulia
ICD-9-CM: International Classification of Diseases ninth revision clinical modification
MRI: Magnetic resonance imaging
MV: Mechanical ventilation
NIV: Non invasive ventilation
