# Supplementary material for: Hospitalizations due to respiratory failure in patients with Amyotrophic Lateral Sclerosis and their impact on survival: a population-based cohort study
Source: BMC Pulm Med. 2016 Nov 3;16:136. doi: 10.1186/s12890-016-0297-y (PMC5094098; doi:10.1186/s12890-016-0297-y)
Supplement: Additional file 1: Table S1. — Number and distribution of discharge diagnoses by type of admission, in hospitalizations ending with death. Table S2. Relation between mortality, hospitalizations for respiratory failure and clinical characteristics. Hazard Ratio (HR), with 95 % Confidence Interval (95 % CI). (DOCX 30 kb) [file 12890_2016_297_MOESM1_ESM.docx]

| Table S1. Number and distribution of discharge diagnoses by type of admission, in hospitalizations ending with death. | | | | | | | | | | | | | | | | | | | | |
| --- | --- | --- | --- | --- | --- | --- | --- | --- | --- | --- | --- | --- | --- | --- | --- | --- | --- | --- | --- | --- |
|  | | Type of admission | | | | | | | | | | | | | | |  | | | |
|  | | Emergency  (N = 97) | | | |  | Elective  (N=16) | | | |  | Day Hospital  (N = 1) | | | |  | Total  (N=114) | | | |
|  | |  |  |  |  |  |  |  |  |  |  |  |  |  |  |  |  |  |  |  |
| Discharge diagnosis  (ICD-9-CM codes) | | Primary code^1^ | | Any  code^2^ | |  | Primary code^1^ | | Any  code^2^ | |  | Primary code^1^ | | Any  code^2^ | |  | Primary code^1^ | | Any  code^2^ | |
|  |  | N | % | N | % |  | N | % | N | % |  | N | % | N | % |  | N | % | N | % |
| ALS (335.20) | | 40 | 41.2 | 85 | 87.6 |  | 1 | 31.2 | 11 | 68.7 |  | 1 | 100.0 | 1 | 100.0 |  | 46 | 40.3 | 97 | 85.0 |
| respiratory failure (518.81-518.84) | | 21 | 21.6 | 56 | 57.7 |  | 5 | 31.2 | 11 | 68.7 |  | - | - | - | - |  | 26 | 22.8 | 67 | 58.8 |
|  | acute (518.81) | - | - | 38 | 39.1 |  | - | - | 10 | 62.5 |  | - | - | - | - |  | - | - | 49 | 42.9 |
|  | chronic (518.83) | - | - | - | - |  | - | - | 1 | 6.2 |  | - | - | - | - |  | - | - | 1 | 0.8 |
|  | acute on chronic (518.84) | - | - | 8 | 8.2 |  | - | - | - | - |  | - | - | - | - |  | - | - | 8 | 7.0 |
|  | unidentified (518.82) | - | - | 10 | 10.3 |  | - | - | - | - |  | - | - | - | - |  | - | - | 10 | 8.7 |
| diseases of respiratory system (460-519) | | 14 | 14.4 | 43 | 44.3 |  | - | - | 7 | 43.7 |  | - | - | - | - |  | 14 | 12.2 | 50 | 43.9 |
|  | pneumonia and influenza (480-487) | - | - | 31 | 31.9 |  | - | - | 6 | 37.5 |  | - | - | - | - |  | - | - | 37 | 32.4 |
|  | aspiration pneumonia (507.0) | - | - | 4 | 4.1 |  | - | - | 1 | 6.2 |  | - | - | - | - |  | - | - | 5 | 4.3 |
| diseases of circulatory system (390-459) | | 5 | 5.1 | 39 | 40.2 |  | 1 | 6.2 | 3 | 18.7 |  | - | .- | 1 | 100.0 |  | 6 | 5.2 | 43 | 37.7 |
| symptoms, signs, ill-defined conditions (780-799) | | 1 | 1.0 | 32 | 32.9 |  | 2 | 12.5 | 4 | 25.0 |  | - | - | - | - |  | 3 | 2.6 | 36 | 31.5 |
| endocrine, nutritional and metabolic diseases, and immunity disorders (240-279) | | 1 | 1.0 | 14 | 14.4 |  | - | - | 2 | 12.5 |  | - | - | - | - |  | 1 | 0.8 | 16 | 14.0 |
| diseases of nervous system and sense organs (320-389) | | 3 | 3.0 | 8 | 8.2 |  | 2 | 12.5 | 5 | 31.2 |  | - | - | - | - |  | 5 | 4.3 | 13 | 11.4 |
| diseases of digestive system  (520-579) | | 2 | 2.0 | 5 | 5.1 |  | 1 | 6.2 | 1 | 6.2 |  | - | - | - | - |  | 3 | 2.6 | 6 | 5.2 |
| diseases of musculoskeletal system and connective tissue (710-739) | | - | - | 5 | 5.1 |  | - | - | - | - |  | - | - | - | - |  | - | - | 5 | 4.3 |
| infectious and parasitic diseases (001-139) | | 1 | 1.0 | 3 | 3.0 |  | - | - | 2 | 12.5 |  | - | - | - | - |  | 1 | 0.8 | 5 | 4.3 |
| diseases of genitourinary system (580-629) | | 1 | 1.0 | 3 | 3.0 |  | - | - | 1 | 6.2 |  | - | - | - | - |  | 1 | 0.8 | 4 | 3.5 |
| injury and poisoning (800-999) | | - | - | 2 | 2.0 |  | - | - | 2 | 12.5 |  | - | - | - | - |  | - | - | 4 | 3.5 |
| neoplasms (140-239) | | 1 | 1.0 | 2 | 2.0 |  | - | - | - | - |  | - | - | - | - |  | 1 | 0.8 | 2 | 1.7 |
| mental disorders (290-319) | | - | - | 2 | 2.0 |  | - | - | - | - |  | - | - | - | - |  | - | - | 2 | 1.7 |
| factors influencing health status and contact with health services (v01-v82) | | 7 | 7.2 | - | - |  | - | - | - | - |  | - | - | - | - |  | 7 | 6.1 | - | - |
| ^1^ Number and percentage (%) of hospitalizations with the corresponding discharge diagnosis as primary code.  ^2^ Number and percentage (%) of hospitalizations with the corresponding discharge diagnosis as primary or any secondary code. | | | | | | | | | | | | | | | | | | | | |

| Table S2. Relation between mortality, hospitalizations for respiratory failure and clinical characteristics. Hazard Ratio (HR), with 95% Confidence Interval (95%CI). | | | | | | | | | | | | | | | | |
| --- | --- | --- | --- | --- | --- | --- | --- | --- | --- | --- | --- | --- | --- | --- | --- | --- |
|  | | Univariate | | |  | Multivariate^1^ | | |  | Multivariate^2^ | | |  | Multivariate^3^ | | |
|  | | p | HR | 95%CI |  | p | HR | 95%CI |  | p | HR | 95%CI |  | p | HR | 95%CI |
| Hospitalization for respiratory failure after ALS diagnosis | |  |  |  |  |  |  |  |  |  |  |  |  |  |  |  |
|  | No^4^ |  | 1.00 | - - |  |  | 1.00 | - - |  |  | 1.00 | - - |  |  | 1.00 | - - |
|  | Yes | <0.0001 | 3.52 | 2.65; 4.67 |  | <0.0001 | 4.53 | 3.35; 6.12 |  | <0.0001 | 4.30 | 3.22; 5.75 |  | <0.0001 | 4.00 | 3.00; 5.34 |
| Site of onset | |  |  |  |  |  |  |  |  |  |  |  |  |  |  |  |
|  | spinal^4^ |  | 1.00 | - - |  |  | 1.00 | - - |  |  | 1.00 | - - |  |  | 1.00 | - - |
|  | bulbar | 0.0005 | 1.71 | 1.27; 2.32 |  | 0.1346 | 1.28 | 0.93; 1.77 |  | 0.1413 | 1.27 | 0.92; 1.75 |  | 0.0791 | 1.32 | 0.97; 1.81 |
|  | missing | 0.0084 | 1.69 | 1.14; 2.49 |  | 0.2243 | 1.32 | 0.84; 2.05 |  | 0.1615 | 1.37 | 0.09; 2.11 |  | 0.2113 | 1.31 | 0.86; 2.01 |
| Age (years) | |  |  |  |  |  |  |  |  |  |  |  |  |  |  |  |
|  | <68^4^ |  | 1.00 | - - |  |  | 1.00 | - - |  |  | 1.00 | - - |  |  | 1.00 | - - |
|  | ≥68 | <0.0001 | 2.05 | 1.56; 2.69 |  | <0.0001 | 2.94 | 2.12; 4.08 |  | <0.0001 | 2.72 | 2.01; 3.70 |  | <0.0001 | 2.50 | 1.86; 3.36 |
| Delay to diagnosis | |  |  |  |  |  |  |  |  |  |  |  |  |  |  |  |
|  | ≥ median^4, 5^ |  | 1.00 | - - |  |  | 1.00 | - - |  |  | 1.00 | - - |  |  | 1.00 | - - |
|  | < median^5^ | 0.0065 | 1.81 | 1.18; 2.77 |  | <0.0001 | 2.58 | 1.65; 4.05 |  | <0.0001 | 2.53 | 1.62; 3.97 |  | 0.0004 | 2.23 | 1.43; 3.47 |
|  | unknown | 0.1095 | 1.34 | 0.94; 1.91 |  | 0.0042 | 1.80 | 1.20; 2.69 |  | 0.0059 | 1.76 | 1.18; 2.63 |  | 0.0459 | 1.48 | 1.01; 2.19 |
| Charlson Index | |  |  |  |  |  |  |  |  |  |  |  |  |  |  |  |
|  | 0^4^ |  | 1.00 | - - |  |  | 1.00 | - - |  |  | 1.00 | - - |  | - | - | - - |
|  | 1-2 | 0.8568 | 0.97 | 0.72; 1.31 |  | 0.0704 | 0.74 | 0.54; 1.02 |  | 0.0869 | 0.76 | 0.55; 1.04 |  | - | - | - - |
|  | ≥ 3 | 0.3208 | 0.82 | 0.55; 1.22 |  | 0.0023 | 0.50 | 0.32; 0.78 |  | 0.0005 | 0.48 | 0.31; 0.72 |  | - | - | - - |
| Sex | |  |  |  |  |  |  |  |  |  |  |  |  |  |  |  |
|  | Men^4^ |  | 1.00 | - - |  |  | 1.00 | - - |  | - | - | - - |  | - | - | - - |
|  | Women | 0.0720 | 1.28 | 0.98; 1.69 |  | 0.2988 | 0.85 | 0.63; 1.15 |  | - | - | - - |  | - | - | - - |
| Hospitalization for respiratory failure before ALS diagnosis | |  |  |  |  |  |  |  |  |  |  |  |  |  |  |  |
|  | No^4^ |  | 1.00 | - - |  |  | 1.00 | - - |  | - | - | - - |  | - | - | - - |
|  | Yes | 0.0871 | 1.68 | 0.93; 3.03 |  | 0.3783 | 0.74 | 0.37; 1.46 |  | - | - | - - |  | - | - | - - |
| ^1^ Includes terms for site of onset, age, lag time to diagnosis, sex, hospitalization for RF before ALS diagnosis, Charlton Index.  ^2^ Includes terms for site of onset, age, lag time to diagnosis, Charlson Index.  ^3^ Includes terms for site of onset, age, lag time to diagnosis.  ^4^ Reference category.  ^5^ Median: 276 days. | | | | | | | | | | | | | | | | |
